# Description of Symptoms Caused by the Infection of the SARS-CoV-2 B.1.621 (Mu) Variant in Patients With Complete CoronaVac Vaccination Scheme: First Case Report From Santiago of Chile

**DOI:** 10.3389/fpubh.2022.797569

**Published:** 2022-03-21

**Authors:** Carlos Barrera-Avalos, Roberto Luraschi, Claudio Acuña-Castillo, Mabel Vidal, Andrea Mella-Torres, Ailen Inostroza-Molina, Rodrigo Vera, Sergio Vargas, Iván Hernández, Christian Perez, Eva Vallejos-Vidal, Daniel Valdés, Mónica Imarai, Felipe E. Reyes-López, Ana María Sandino

**Affiliations:** ^1^Centro de Biotecnología Acuícola, Facultad de Química y Biología, Universidad de Santiago de Chile, Santiago, Chile; ^2^Department of Biology, Faculty of Chemistry and Biology, University of Santiago de Chile, Santiago, Chile; ^3^Department of Computer Science, University of Concepcion, Concepción, Chile; ^4^Hospital de Urgencia Asistencia Pública (HUAP), Santiago, Chile; ^5^Centro de Nanociencia y Nanotecnología CEDENNA, Universidad de Santiago de Chile, Santiago, Chile; ^6^Facultad de Medicina Veterinaria y Agronomía, Universidad de Las Américas, Santiago, Chile; ^7^Department of Cell Biology, Physiology and Immunology, Universitat Autònoma de Barcelona, Bellaterra, Spain

**Keywords:** SARS-CoV-2 B.1.621 variant, genomic surveillance, inactivated SARS-CoV-2 vaccine, CoronaVac, case report, symptoms, COVID-19

## Abstract

Vaccine administration is one of the most efficient ways to control the current coronavirus disease 2019 (COVID-19) pandemic. However, the appearance of severe acute respiratory syndrome coronavirus 2 (SARS-CoV-2) variants can avoid the immunity generated by vaccines. Thus, in patients with a complete vaccine schedule, the infection by SARS-CoV-2 may cause severe, mild, and asymptomatic manifestations of the disease. In this case report, we describe for the first time the clinical symptoms of four patients (three symptomatic; one asymptomatic) from Santiago of Chile, with a complete vaccination schedule with two doses of CoronaVac (Sinovac Life Science) infected with the variant of interest (VOI) B.1.621 (Mu). They were compared with four unvaccinated patients, who had a higher prevalence of symptoms after infection compared to vaccinated patients. In the CoronaVac-vaccinated group, an 80-year-old patient who registered various comorbidities required Invasive mechanical ventilation for 28 days with current home medical recovery discharge. By contrast, in the unvaccinated group, a 71-year-old presented more symptoms with more than 45 days of Invasive mechanical ventilation, which continues to date, presenting greater lung damage than the vaccinated hospitalized patient. This first report evidence differences in the clinical symptomatology of patients vaccinated and non-vaccinated infected with the VOI B.1.621 (Mu) and suggest the protective effects of CoronaVac against this variant.

## Introduction

The coronavirus disease 2019 (COVID-19) pandemic has left more than 4.8 million deaths around the world and 240 million infections to date. Therefore, the health authorities of all countries have implemented various protocols to prevent its spread. In this way, massive vaccination has been the most effective strategy to reduce the number of positive cases in the population and the severe clinical manifestations of the disease (1–3). However, the appearance of new variants of severe acute respiratory syndrome coronavirus 2 (SARS-CoV-2) could evade the immune protection of people with a completed vaccination scheme and manifest as symptomatic disease. Hacisuleyman et al., reported that SARS-CoV-2 variants can reinfect patients and generate new symptomatologic manifestations of the disease (4), while asymptomatic cases may decrease even up to 65% in some study populations with B.1.1.7 (Alpha) variant (5). Therefore, the prevalence and severity of the symptoms generated by the different SARS-CoV-2 variants in a scenario of a complete vaccination scheme are of great scientific and public interest. In this report, we describe for the first time the clinical symptoms of patients infected with the new variant of interest (VOI) B.1.621 (Mu) under a complete vaccination scheme with the inactivated virus vaccine CoronaVac from Sinovac Life Sciences, the widest immunization strategy administered in Chile (6). The WHO listed the Mu as a VOI on August 31, 2021 (7) and shared mutations with variants of concern (VOC) (8). The B.1.621 variant could evade neutralizing antibodies in patients fully vaccinated with the BNT162b2 (Pfizer/BioNTech) vaccine (9) and is 10 times more resistant to neutralizing antibodies than the ancestral strain (10). Therefore, it is interesting to increase the knowledge about the Mu variant spreading in countries following a different vaccination scheme schedule. In this scenario, currently, there is no information available regarding the clinical manifestations of Mu variant infection versus a complete vaccination schedule with the CoronaVac vaccine. In this case report, we detected fewer symptoms in patients fully vaccinated with CoronaVac compared to the unvaccinated. Furthermore, between two hospitalized patients, we observed that the vaccinated patient, even having four comorbidities and 80 years, recovered faster compared to the 71-year-old unvaccinated patient with only one comorbidity, who Regrettably is still hospitalized. Our report reinforces the relevance of public health policies aimed to massify the vaccination strategy in the population and the differences in the manifestation of disease in people fully immunized with CoronaVac schedule against new viral variants.

## Cases Description

**Figure 1:**
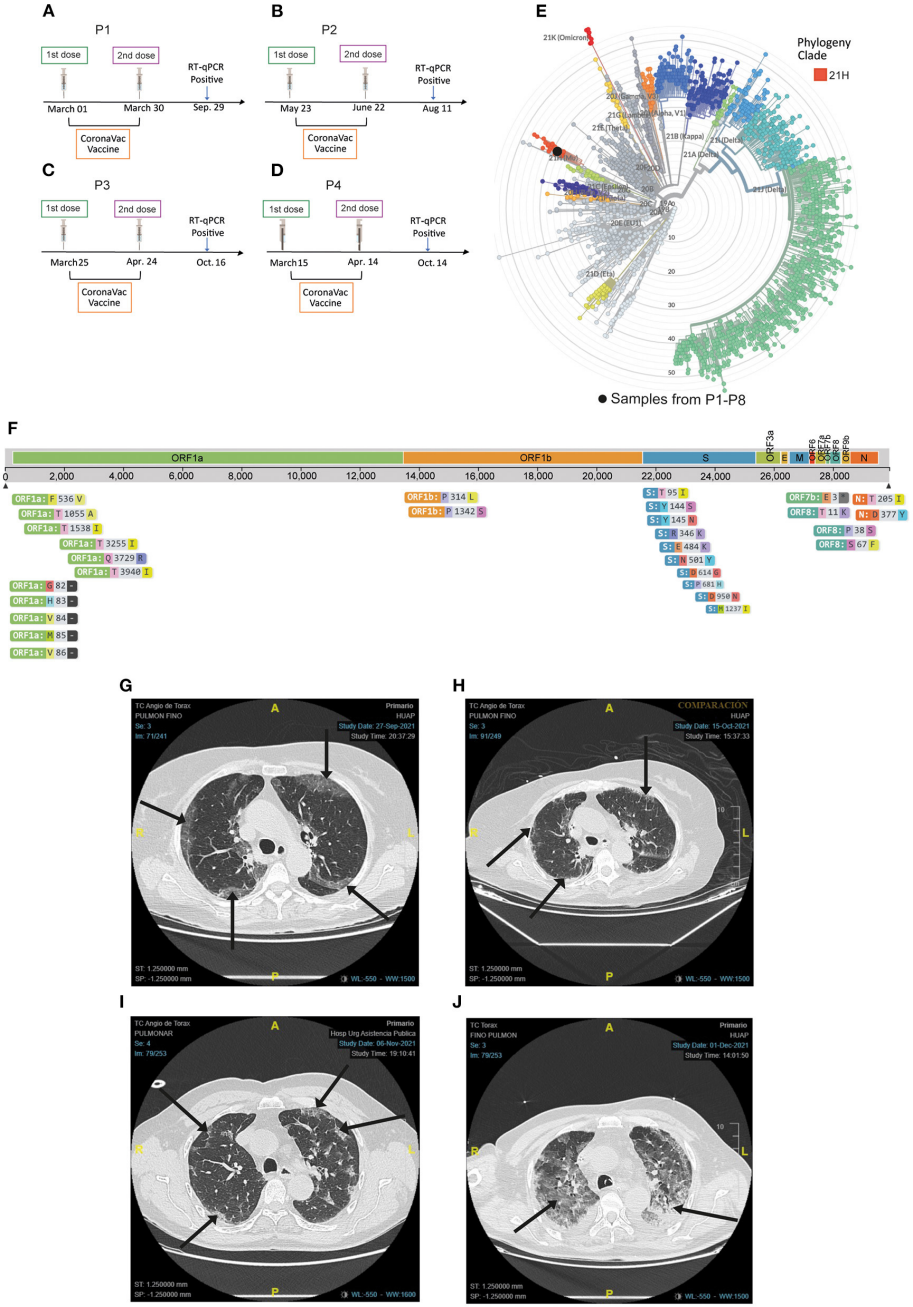
Representative SARS-CoV-2 genome sequencing of the nasopharyngeal swab samples (NPSs) and severity of infection in hospitalized patients. **(A–D)** Timeline of vaccination scheme from P1, P2, P3, and P4, respectively. **(E)** Representative phylogenetic tree placement of all NPSs using Nextstrain clades classification, all samples belong to clade 21H. **(F)** The sequence was aligned with a representation of the global SARS-CoV-2 genetic diversity and amino acid substitutions identified in the SARS-CoV-2 Mu sequence are shown. **(G–J)** Representative chest computed angio-tomography (CCA-T) of patients hospitalized with IVM. **(G)** Image of the 80-year-old patient (P1) vaccinated with CoronaVac. Ground-glass opacity (GGO) and 32.88% of pulmonary involvement are shown, upon admission to the hospital emergency department (September 27). **(H)** CCA-T is shown 19 days after the first CCA-T, after a cycle of IMV, with pulmonary involvement and recovery of 17.69%. **(I)** Image of the 71-year-old patient (P7) unvaccinated. GGO and 18.59% lung involvement are shown upon admission to the hospital emergency department (November 6). **(J)** CCA-T is shown 26 days after the first CCA-T after a cycle of IMV. GGO is shown with a pulmonary involvement of 68.63%. All black arrows indicate GGO. All images were obtained from similar focal planes between both patients.

The first case of symptomatology description includes four patients with complete vaccination schedule (two doses) of CoronaVac (Sinovac Life Science) infected with the VOI Mu. The data describe two patients who are men and aged 27 and 33 years old, and two patients who are women and aged 35 and 80 years old. They become COVID-19 positive after 175, 183, 50, and 183, days respectively after the second dose of vaccination (Figures 1A–D). The second group describes the symptoms of four unvaccinated male patients of 27, 38, 43, and 71 years old infected with the Mu variant as well. The main symptoms and frequent comorbidities associated with the COVID-19 disease are described according to the Centers for Disease Control and Prevention (CDC) (11, 12). The nasopharyngeal swab samples (NPSs) for COVID-19 diagnosis by RT-qPCR were obtained through the massive search strategy for cases carried out by the Chilean Ministry of Health and from patients treated at the Hospital de Urgencia Asistencia Pública (HUAP), Santiago, Chile.

## Diagnostic Assessment

The patients were diagnosed for COVID-19 using the total RNA extracted from NPSs through the amplification of SARS-CoV-2 ORF1ab and N gene by RT-qPCR (TaqMan™ 2019nCoV Assay Kit v1, Thermo Fisher Scientific, Massachusetts, USA. Cat. No. A47532 and E, N, and RdRp gene for Allplex™ 2019-nCoV Assay, Seegene, Seúl, South Korea. Cat, No RP10244Y) as previously reported by our group (13) and other authors (14, 15). The detection of virus-specific mutations by Real-Time RT-PCR kit (Bioneer, Daejeon, South Korea. Cat. No. SMVR-2112 and Allplex™ SARS-CoV-2 Variants I Assay, Seegene Company, Seúl, South Korea) was made following manufacturer instructions. In the Seegene kit, the presence of E484K and N501Y suggested the presence of Mu variant, while Bioneer kit, the N501Y, E484K, P681H mutations. All samples were confirmed by NGS at the Public Health Institute of Chile (ISP, for her Spanish acronym) and Microbial Genome Sequencing Center laboratory, USA. Representative MiSeq and NextSeq 2000 sequencing confirmed the presence of the Mu variant of SARS-CoV-2, being classified on the Nextstrain Clade 21H (NextClade software version 1.9, Biozentrum, University of Basel, Swiss) (Figure 1E) and following the World Health Organization (WHO) recommendations (16). This variant presented nucleotide diversity, mainly in the ORF1a, S, ORF7a / b, and N genes (Figure 1F), and mutations shared with other variants of concern (VOC) including S: P681H, S: D614G, and S: D950N (8). The sequence analysis also showed the substitutions S:T95I, S: R346K, ORF8:T11K, S: Y144S, and S: Y145N (Figure 1F), Altogether, these substitutions are associated with variant B.1.621 according to the characterization reported by Laiton-Donato et al. (17). From the four patients vaccinated with CoronaVac, three were symptomatic (P1–P3) and one was asymptomatic (P4). All the unvaccinated patients manifested symptoms of disease (Table 1). In addition, the highest prevalence of reported symptoms was in unvaccinated patients, with dry cough, and muscle or body aches in all patients (Table 1). On the other hand, in vaccinated patients, the main symptoms associated with the infection were dry cough, nasal congestion, loss of taste, and headache (Table 1). The patients (P1–P8) presented different viral loads at the beginning of the disease. The lowest viral load was detected in unvaccinated P6 (2.5 × 10^3^ copies/μl), who presented several symptoms (Table 2). The highest viral load was presented in vaccinated P4 (3.4 × 10^5^ copies/μl), who were asymptomatic (Table 2). Patient P2 reported no other positive case among her social circle (close contact, work colleagues, and family). This patient did not report RT-qPCR at the end of his quarantine. For the other patients, there is no follow-up information available at the end of the symptoms.

**Table 1:** Summary of symptom prevalence and severity in CoronaVac vaccinated and unvaccinated patients (n°).

	**CoronaVac**	**Unvaccinated**
Symptomatic	3	4
Asymptomatic	1	0
Age range	27–80	27–71
**Symptoms**
Fever or chills	0	2
Dry cough	2	3
Cough w/expectoration	1	1
Shortness of breath or difficulty breathing	1	0
Fatigue	0	0
Muscle or body aches	0	4
Loss of smell	1	1
Headache	2	2
Loss of taste	2	1
Sore throat	1	1
Congestion or runny nose	2	1
Lumbar pain	0	2
Nausea or vomiting	0	1
Diarrhea	0	1
**Total symptoms**	12	19
**Hospitalization**
Mechanic ventilation	1	1

**Table 2:** Specific symptomatology and clinical records reported by B.1.621 (Mu) variant infection in full vaccinated with CoronaVac and unvaccinated patients.

	**CoronaVac**				**Unvaccinated**			
	**P1**	**P2**	**P3**	**P4**	**P5**	**P6**	**P7**	**P8**
Age	80	35	27	33	38	27	71	43
Gender	F	F	M	M	M	M	M	M
Symptomatic	✓	✓	✓	X	✓	✓	✓	✓
Asymptomatic	X	X	X	✓	X	X	X	X
Days after the 2nd dose^*^	183	50	175	183	Not applicable			
**Symptoms**
Fever or chills	X	X	X	X	X	X	✓	✓
Dry cough	X	✓	✓	X	✓	✓	✓	X
Cough w/expectoration	✓	X	X	X	X	X	X	✓
Shortness of breath or difficulty breathing	✓	X	X	X	X	X	X	X
Fatigue	X	X	X	X	X	X	X	X
Muscle or body aches	X	X	X	X	✓	✓	✓	✓
Loss of smell	X	✓	X	X	X	✓	X	X
Headache	X	✓	✓	X	X	✓	X	✓
Loss of taste	X	✓	✓	X	X	✓	X	X
Sore throat	X	X	✓	X	X	X	X	✓
Congestion or runny nose	X	✓	✓	X	X	X	✓	X
Lumbar pain	X	X	X	X	✓	X	X	✓
Nausea or vomiting	X	X	X	X	X	X	X	✓
Diarrhea	X	X	X	X	X	X	X	✓
**Comorbidities**
Diabetes mellitus II	✓	X	X	X	X	X	X	X
Arterial hypertension	✓	X	X	✓	X	X	✓	X
Diabetes mellitus I	X	X	X	X	X	✓	X	X
Hypothyroidism	✓	X	X	X	X	X	X	X
Mitral valve disease	✓	X	X	X	X	X	X	X
**Hospitalization**
Mechanic ventilation	✓^**^	X	X	X	X	X	✓^#^	X
**Viral load**
(Viral copies/μl)	7.1 × 10^4^	9.0 × 10^4^	1.2 × 10^5^	3.4 × 10^5^	1.4 × 10^5^	2.5 × 10^3^	5.2 × 10^4^	4.8 × 10^4^

* *Elapsed time from the 2nd dose to the day positive COVID-19 diagnosis.*

** *Currently home care monitoring.*

# *Currently in oxygen administration at HUAP.*

✓ *presence;*

X *absence.*

*In the “Symptoms” section, the gray color highlights the presence of symptoms*.

A vaccinated 80-year-old patient (P1) with comorbidities, such as Diabetes type 1 (DM1), hypothyroidism, arterial hypertension, and mitral valve disease was hospitalized and admitted into the intensive care unit (ICU) for 48 days with 28 days of invasive mechanical ventilation (IMV). Her symptoms were just coughing with expectoration and shortness of breath (Table 2). She is currently without IMV at-home care assistance and monitoring. The first chest computed angio-tomography (CCA-T) was done at admission in the emergency department of HUAP, Chile on September 27, 2021, showing predominantly subpleural bilateral ground-glass opacity (GGO) with 32% pulmonary area involvement (Figure 1G). The patient was admitted to ICU and intubated with IMV on September 30, 2021. On October 1, a second CCA-T was performed, and the lung involvement decreased to 19.55% (Table 3). On October 9, she was extubated, and 6 days later a third CCA-T was performed, showing minimal GGO patches, basal subpleural consolidations with a pulmonary involvement of 17.69% (Figure 1H). On October 16, she was re-intubated with IMV due to bronchial aspiration. On October 31, a fourth CCA-T was performed, with 18.41% pulmonary involvement (Table 3). The patient was extubated on November 3, and a fifth CCA-T was performed on November 21, even with pulmonary involvement and detection of GGO of the upper lobes (Table 3). On December 7, the last CCA-T showed regression and residual GGO as post-COVID-19 results. The patient was discharged with home monitoring on December 12. In Figures 1G,H, only CCA-T representative of the initial state (September 27) and during the infection (October 15) by COVID-19 disease are shown.

**Table 3:** Progression of COVID-19 disease in vaccinated (P1) and non-vaccinated (P7) patients requiring invasive mechanical ventilation.

	**P1**	**P7**
Symptom onset	September 18, 2021	October 28, 2021
Admission to the emergency department	September 27, 2021	November 6, 2021
1st Chest Computed Angio- Tomography (CCA-T)	September 27, 2021: 32.88% pulmonary involvement	November 6, 2021: 18.59% pulmonary involvement
1st intubation with invasive mechanical ventilation (IMV)	September 30, 2021	November 8, 2021
2nd CCA-T	October 01, 2021: 19.55% pulmonary involvement	November 19, 2021: 48.10% pulmonary involvement
1st Extubation	October 9, 2021	November 24, 2021
3rd CCA-T	October 15, 2021: 17.69% pulmonary involvement	December 1, 2021: 68.3% pulmonary involvement, and laminar bilateral pleural effusion
Complications	Bronchial aspiration	Lung rigidity
Re-intubation with IMV	October 16, 2021	November 30, 2021
4th CCA-T	October 31, 2021: 18.41% pulmonary involvement	December 15, 2021: Extensive lung involvement of 60.04% in ground-glass opacity (GGO). Bilateral pleural effusion
2nd Extubatioon	November 3, 2021	Not applicable
5th CCA-T	November 21, 2021: Extensive upper lobe involvement in predominantly left ground-glass opacity (GGO)	Not applicable
6th CCA-T	Regression and residual GGO lattice images	Not applicable
Medical discharge	December 12, 2021: hospitalization and home monitoring	Current hospitalization with IMV (January 10, 2022)

On the other hand, in the unvaccinated group, we registered a 71-year-old patient (P7) with arterial hypertension, who presented fever, dry cough, body aches, and nasal congestion. The patient remains hospitalized until the date of this report, with 58 days in the ICU and 45 days of IMV. He was admitted to the emergency department on November 6, 2021, a CCA-T was performed, showing bilateral, subpleural, and greater GGO toward the bases, which involved 18.59% of the lungs (Figure 1I). The patient was intubated with IMV on November 8, and a second CCA-T was performed on November 19, with identification of GGO and condensed areas with lung involvement of 48.1% (Table 3). He was extubated on November 24 with a high-flow basal cannula (H-FBC) for 2 days. On November 26, the patient was subjected to Non-invasive mechanical ventilation (NIMV) until November 30, when he was re-intubated due to high pulmonary rigidity (Table 3). On December 1, he was subjected to a third CCA-T, showing extensive areas of GGO and laminar bilateral pleural effusion with 68.63% pulmonary involvement (Figure 1J). The patient undergoes a prone cycle between December 6 and 8. The last CCA-T was performed on December 15, maintaining extensive areas of GGO and bilateral pleural effusion with 60.04% pulmonary involvement (Table 3). Until January 11, he remained in hospitalization with nasal oxygen administration.

## Discussion

The current application of different COVID-19 vaccines has made it possible to reduce the spreading of the infection and severity of the disease in several countries (3). However, this protection has been threatened by the new variants of the virus originally described in Wuhan, China (18). The decrease in the protection effectiveness of vaccines can be related to a slight increase in symptoms against infections by SARS-CoV-2 variants like in B.1.617.2 (Delta) (19) and sometimes developing severe disease scenarios (20). Therefore, the symptomatology of COVID-19 disease caused by some variants in vaccinated patients is a matter of great public interest and concern.

In Chile, the most administered vaccine is the inactivated-virus-CoronaVac (Sinovac Life Sciences), approved by the ISP for emergency use (20). A phase 3 trial reported by Jara et al., demonstrates the effectiveness of 65.9% against SARS-CoV-2 infection, while its capacity for hospitalizations prevention was 87.5% (21). A study carried out in São Paulo, Brazil, in a cohort of people >70 years old, indicated that the percentage of hospitalizations after a complete vaccination scheme with CoronaVac was 77.6% for VOC P.1 (Gamma) (22), suggesting greater symptoms compared to SARS-CoV-2 Wuhan. However, there are no reports of symptoms associated with Mu variant in patients with a complete CoronaVac vaccine scheme. The Mu variant was cataloged as VOI by the WHO on August 31, 2021(7). Its mutations panel may suggest greater spread, immune escape, and, therefore, a more severe situation of the COVID-19 disease (15, 16). Thus, full vaccinated patients with CoronaVac and the development of disease in those infected cases is an important and unexplored focus of interest and study.

In this report, we describe for the first time the symptomatologic scenario of four patients infected with Mu variant after 172 (P1), 50 (P2), 171 (P3), and 183 (P4) days of completing their two-doses vaccination schedule with CoronaVac vaccine. The patients P2 and P3 did not report comorbidities but reported five mild symptoms, while the P4 was asymptomatic even having the highest viral load of all patients and with a clinical history of arterial hypertension, the most common COVID-19 comorbidity that increased risk of severe disease (23). The unvaccinated P8 patient also does not present comorbidities but reports at least eight symptoms related to the disease, including cough with expectoration, a sign of severe infection (24). Imaging examination of hospitalized patients (P1: vaccinated with CoronaVac; P7: unvaccinated) show greater pulmonary involvement in the unvaccinated patient, who unfortunately remains on IMV due to the infection by Mu variant to date of this report. Although age affects the immune response (25), in our study we observe differences in disease progression between P1 and P7. Finally, fewer symptoms and greater comorbidity were reported by P1 who was discharged from the hospital with home care monitoring. The patient P7 presented more symptoms, fewer comorbidities, and greater pulmonary involvement by Mu infection in the setting of severe illness. These data suggest a protective effect relationship of the CoronaVac vaccine against the Mu variant and the severe manifestation of disease. This could be related to other reports that indicate, for example, that an infection by VOC B.1.1.7 (Alpha) in full vaccinates induces asymptomatic scenes or mild symptoms (26). Other variants such as Gamma can also cause hospitalizations in patients fully vaccinated with CoronaVac (27). On the other hand, Messali et al., reported that the protection against Mu variant generated by other vaccines following a RNA immunization strategy like the BNT162b2 vaccine (Pfizer/BioNTech) could be significantly lower than the generated against the original SARS-CoV-2 virus (9), a situation that could also happen for the case of other vaccines, like CoronaVac.

This is a descriptive report of the Mu variant infection in patients with a complete CoronaVac vaccine schedule. However, the information is still limited to conclude that mild symptoms are because of the complete vaccination of the patient. We suggest a direct effect of this vaccine, but it is necessary to increase the number of cases. In this way, the genetic, age, and comorbidities (among other factors of relevance) of the patients, and their potential association to the severity of the disease is also a matter of interest to be considered. In any case, our data are of great interest for future clinical investigations related to CoronaVac, its effectiveness against the new variants of SARS-CoV-2, and the direction of future public health policies aimed at eradicating the pandemic.

## Data Availability Statement

The datasets presented in this study can be found in online repositories. The names of the repository/repositories and accession number(s) can be found at: https://www.ncbi.nlm.nih.gov/, PRJNA772359.

## Ethics Statement

The studies involving human participants were reviewed and approved by Ethical Committee of the University of Santiago of Chile (No. 226/2021) and the Scientific Ethical Committee of the Central Metropolitan Health Service, Ministry of Health, Government of Chile (No. 370/2021). Written informed consent for participation was not required for this study in accordance with the national legislation and the institutional requirements.

## Author Contributions

FER-L, CA-C, AS, EV-V, and MI: conceptualization. FER-L and RL: data curation. IH, CP, RV, SV, and MV: formal analysis. AS: funding acquisition. CB-A, AM-T, and AI-M: investigation. FER-L: methodology. DV: supervision. CB-A and RL: writing—original draft. AMS, FR-L, CB-A, and RV: writing—review and editing. All authors have read and agreed to the published version of the manuscript, contributed to the article, and approved the submitted version.

## Funding

The Laboratory of Virology had the support from the COVID-19 diagnosis in the University Laboratories Network (Ministry of Sciences, Ministry of Health, Government of Chile) for diagnosis tasks. The authors also thank the Rapid Assignment of Resources for Research Projects on the Coronavirus (COVID-19) (project number COVID1038; ANID, Government of Chile), Fondecyt regular project numbers 1201664 (MI) and 1211841 (FER-L) (ANID, Government of Chile), Fondecyt iniciación grant (project number 11221308; ANID, Government of Chile) (EV-V), and DICYT-USACH project number 021943AC (CA-C) grants. The funders had no role in study design, data collection, and analysis, decision to publish, or preparation of the manuscript.

## Conflict of Interest

The authors declare that the research was conducted in the absence of any commercial or financial relationships that could be construed as a potential conflict of interest.

## Publisher's Note

All claims expressed in this article are solely those of the authors and do not necessarily represent those of their affiliated organizations, or those of the publisher, the editors and the reviewers. Any product that may be evaluated in this article, or claim that may be made by its manufacturer, is not guaranteed or endorsed by the publisher.
